# Pancreatic Neuroendocrine Tumor with Acinar Pattern Mimicking Pancreatic Ductal Adenocarcinoma: A Case Report

**DOI:** 10.70352/scrj.cr.26-0184

**Published:** 2026-08-26

**Authors:** Yasunori Shirakawa, Kei Yamane, Kazuyuki Nagai, Shinya Otsuki, Masakazu Fujimoto, Kentaro Tsuji, Tomoaki Yoh, Shinya Okumura, Hirofumi Hirao, Satoshi Ogiso, Takayuki Anazawa, Atsuko Kasajima, Günter Klöppel, Hironori Haga, Etsuro Hatano

**Affiliations:** 1 Department of Surgery, Graduate School of Medicine, Kyoto University, Kyoto, Kyoto, Japan; 2 Department of Surgery, Kobe City Medical Center General Hospital, Kobe, Hyogo, Japan; 3 Department of Diagnostic Pathology, Graduate School of Medicine, Kyoto University, Kyoto, Kyoto, Japan; 4 Department of Surgery, Sapporo Medical University, Sapporo, Hokkaido, Japan; 5 Department of Pathology, Consultation Center for Pancreatic and Endocrine Tumors, TUM School of Medicine and Health, Technical University of Munich, Munich, Germany

**Keywords:** pancreatic neuroendocrine tumor, acinar structure, endoscopic ultrasound-guided tissue acquisition

## Abstract

**INTRODUCTION:**

Pancreatic neuroendocrine tumors (PanNETs) are rare, but their incidence has been increasing. PanNETs are usually diagnosed based on typical radiological and pathological findings. However, some exhibit unusual findings; therefore, we need to consider these and make a diagnosis through multidisciplinary evaluation. In this report, we present a case of PanNET that was challenging to diagnose preoperatively.

**CASE PRESENTATION:**

A man in his 70s with a prior diagnosis of intrahepatic cholangiocarcinoma (iCCA) was referred to our hospital (Kyoto University Hospital) for suspected recurrence of multiple intrahepatic metastases. MRI showed space-occupying lesions in the liver and a 2.7-cm hyperintense mass in the uncinate process of the pancreas. Adenocarcinoma was diagnosed by endoscopic US-guided tissue acquisition (EUS-TA) of the pancreatic lesion. Accordingly, the patient underwent neoadjuvant chemotherapy followed by pancreatoduodenectomy. However, postoperative histological and immunohistochemical evaluations of the surgically resected specimen revealed G2 PanNET with an unusual acinar structure that was not considered preoperatively.

**CONCLUSIONS:**

This case highlights the diagnostic challenge of unusual PanNETs that presented radiologically and pathologically with unusual features. A multidisciplinary evaluation and awareness of PanNETs with unusual features are essential for accurate diagnosis.

## Abbreviations

^18^F-FDG
: fluorine-18 fluorodeoxyglucose
BCL10
: B-cell lymphoma/leukemia 10
CA19-9
: carbohydrate antigen 19-9
CEA
: carcinoembryonic antigen
CECT
: contrast-enhanced CT
CK
: cytokeratin
CRP
: C-reactive protein
DUPAN-2
: duke pancreatic monoclonal antigen type 2
EUS
: endoscopic US
EUS-TA
: endoscopic US-guided tissue acquisition
FNA
: fine needle aspiration
Gd-EOB-DTPA
: gadolinium ethoxybenzyl diethylenetriamine pentaacetic acid
iCCA
: intrahepatic cholangiocarcinoma
NAC-GS
: neoadjuvant chemotherapy with gemcitabine plus S-1
NECs
: neuroendocrine carcinomas
NENs
: neuroendocrine neoplasms
OS
: overall survival
PanNET
: pancreatic neuroendocrine tumor
PDAC
: pancreatic ductal adenocarcinoma
PP
: pancreatic polypeptide
PRRT
: peptide receptor radionuclide therapy
S
: segment
S100P
: S100 calcium-binding protein P
SSA
: somatostatin analog
SSTR2
: somatostatin receptor 2
SUVmax
: maximum standardized uptake value

## INTRODUCTION

PanNETs are rare, accounting for only 1%–2% of pancreatic neoplasms.^1)^ However, the incidence of PanNETs has been increasing, and they are becoming more frequently encountered in daily practice.^2–4)^

PanNETs are diagnosed through multidisciplinary evaluations, including CECT, MRI, and pathological evaluation of biopsy specimens.^5)^ Histopathological diagnosis using EUS-TA plays a crucial role in preoperative diagnosis and treatment planning. Nevertheless, diagnostic accuracy may be limited in some cases due to sampling constraints, tumor heterogeneity, or morphologic overlap with other pancreatic neoplasms. Therefore, a comprehensive assessment that integrates clinical, radiological, and pathological information is essential. Herein, we present a case of a PanNET that was preoperatively diagnosed as PDAC due to unusual acinar histology.

## CASE PRESENTATION

**Fig. 1:**
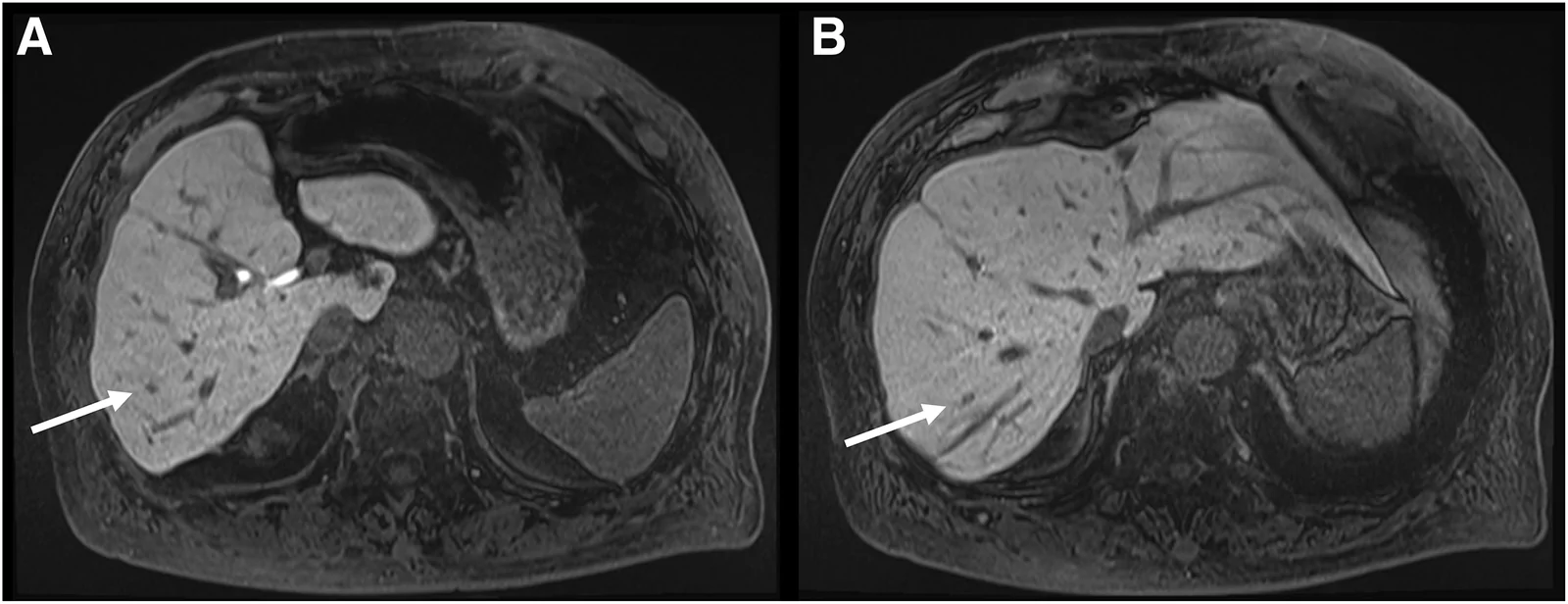
Liver Gd-EOB-DTPA–enhanced MRI in the hepatobiliary phase. (**A**) A space-occupying lesion with no Gd-EOB-DTPA uptake in S6 (arrow). (**B**) A space-occupying lesion with no Gd-EOB-DTPA uptake in S7 (arrow). Gd-EOB-DTPA, gadolinium ethoxybenzyl diethylenetriamine pentaacetic acid; S, segment

**Fig. 2:**
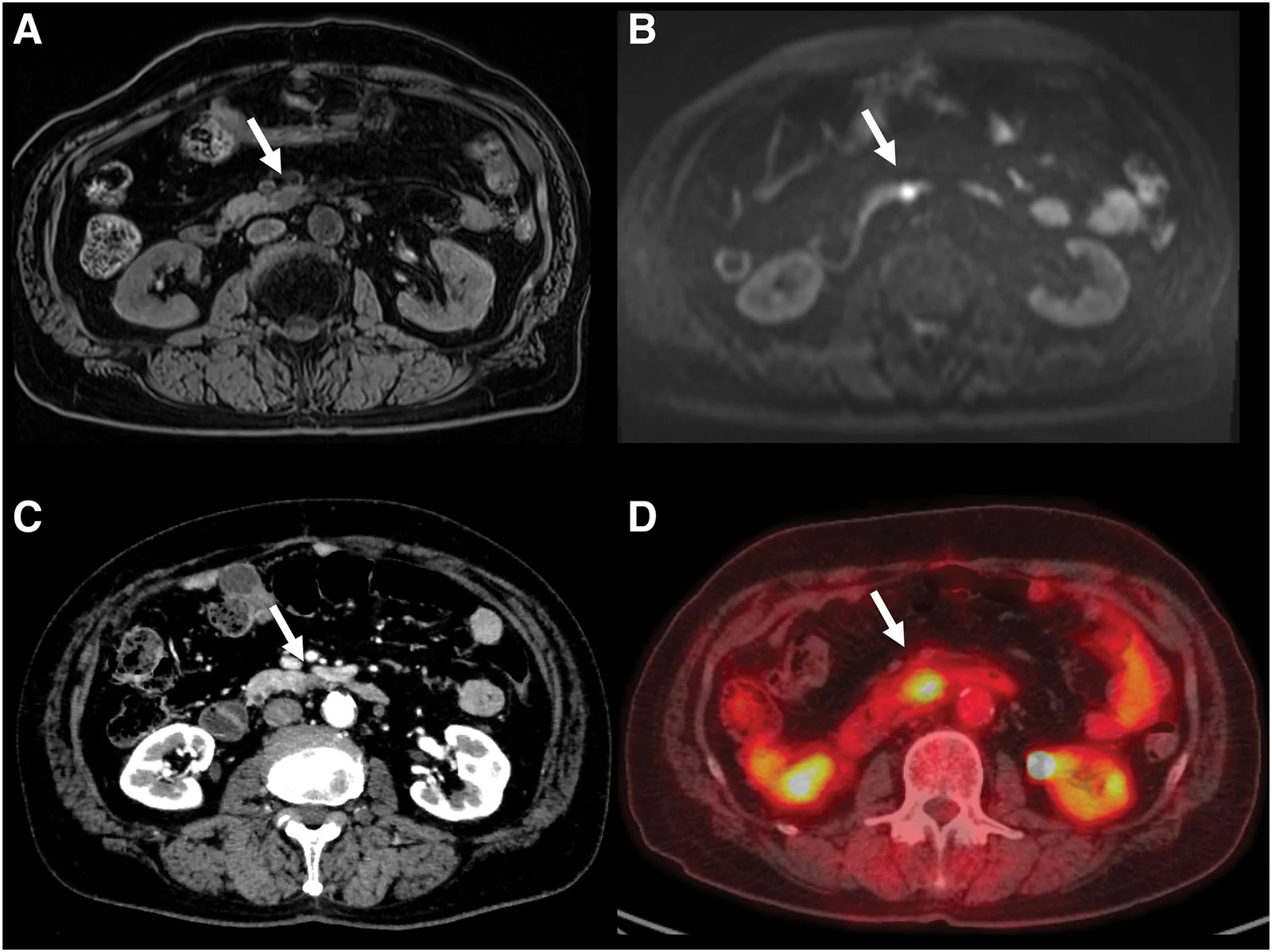
Uncinate process of the pancreas on MRI, CECT, and ^18^F-FDG PET-CT. (**A**) T1-weighted image showing a 2.7-cm hyperintense mass (arrow). (**B**) Diffusion-weighted image showing a hyperintense mass (arrow). (**C**) CECT showing a poorly circumscribed, hypovascular lesion (arrow). (**D**) ^18^F-FDG PET-CT showing FDG uptake (SUVmax 5.0, arrow). ^18^F-FDG, fluorine-18 fluorodeoxyglucose; CECT, contrast-enhanced CT; SUVmax, maximum standardized uptake value

**Fig. 3:**
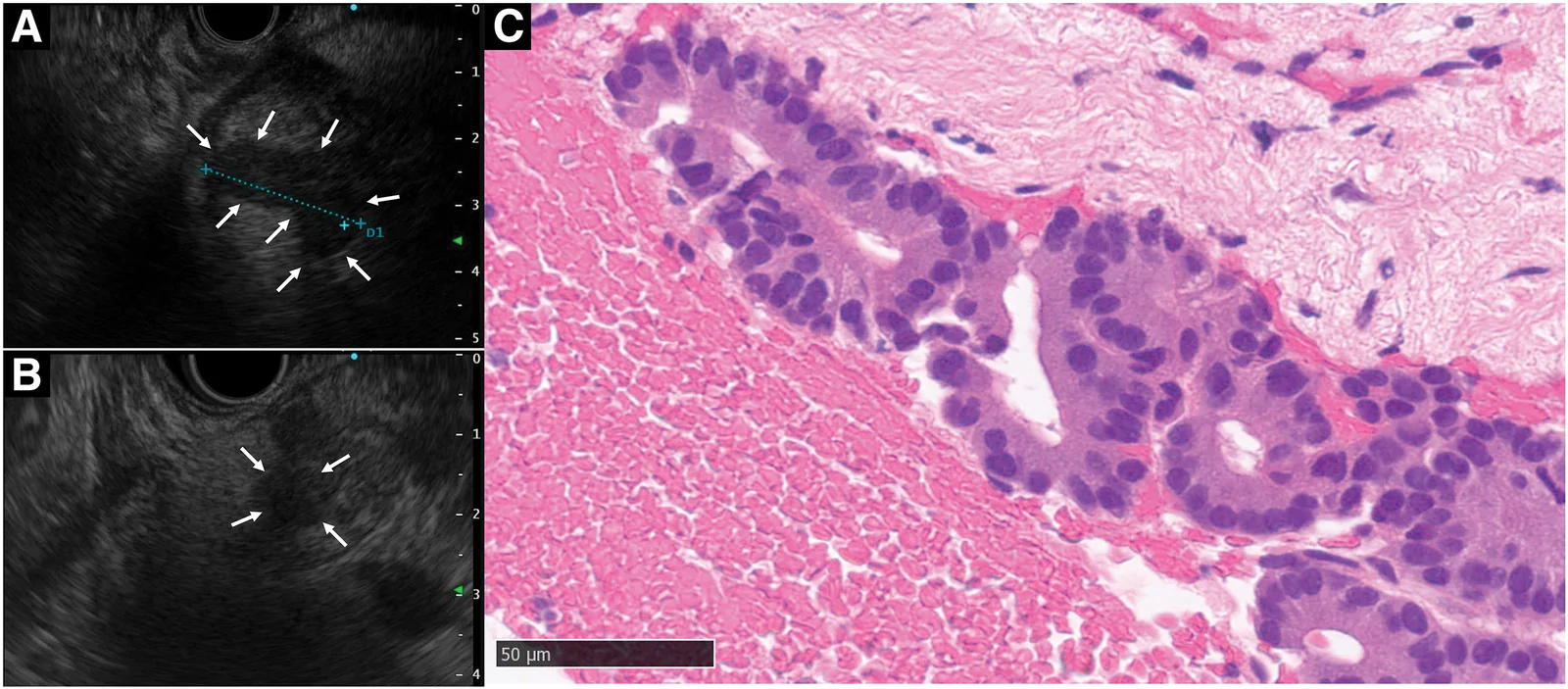
EUS and EUS-TA findings. (**A**) EUS image showing a 25 × 15-mm hypoechoic cystic lesion in the uncinate process (arrows). (**B**) Contrast-enhanced EUS showing no enhancement in the solid components (arrows). (**C**) Neoplastic cells with an acinar pattern observed in EUS-TA of the pancreatic tumor. EUS, endoscopic US; EUS-TA, EUS-guided tissue acquisition

A man in his 70s was referred to our hospital (Kyoto University Hospital) with suspected recurrence of multiple intrahepatic metastases. Three years prior, he had undergone a subsegmentectomy of S5 and limited resections of S3, S6, and S7 at the referring hospital and was diagnosed with iCCA showing multiple intrahepatic metastases. On admission, he was asymptomatic, with normal tumor markers: CEA 3.1 ng/mL, CA19-9 <2.0 U/mL, and DUPAN-2 276 U/mL. Gd-EOB-DTPA–enhanced MRI revealed space-occupying lesions in S6 and S7 (**Fig. 1**). In addition, a 27 × 13-mm hyperintense mass in the uncinate process of the pancreas was detected on both T1-weighted and diffusion-weighted images (**Fig. 2A** and **2B**). The pancreatic mass was poorly circumscribed and hypovascular on CECT and showed FDG uptake on ^18^F-FDG PET-CT (SUVmax 5.0) (**Fig. 2C** and **2D**). EUS identified a 25 × 15-mm hypoechoic cystic lesion in the uncinate process (**Fig. 3A**) and contrast-enhanced EUS showed no enhancement in the solid components (**Fig. 3B**), suggesting either a cystic lesion or a hypovascular solid mass. EUS-TA performed by FNA yielded tissue fragments sufficient for histological and immunohistochemical assessment, although evaluation of the overall tumor architecture was limited by specimen fragmentation. The specimen was composed of epithelial cells arranged in small glandular and acinar structures (**Fig. 3C**). Because the fragmented specimen did not allow a confident assessment of the overall cytologic and architectural uniformity of the lesion, adenocarcinoma was favored. Additional immunohistochemical studies were conducted to evaluate the differential diagnosis between PDAC and iCCA. The results demonstrated negative staining for S100P and CRP and positive staining for CD56. Based on the radiological findings, histological appearance, and patient history, the lesion was interpreted as pancreaticobiliary adenocarcinoma, with metastatic iCCA considered more likely than PDAC. The hepatic lesions were radiologically interpreted as metastases of the previously diagnosed carcinoma of the liver. Overall, the PDAC was classified as resectable, T2N0M0 (Stage IB), according to the Union for International Cancer Control TNM classification, 8th edition. The patient subsequently received 2 cycles of gemcitabine plus S-1 chemotherapy as NAC-GS, followed by discussion in a multidisciplinary conference.^5,6)^

**Fig. 4:**
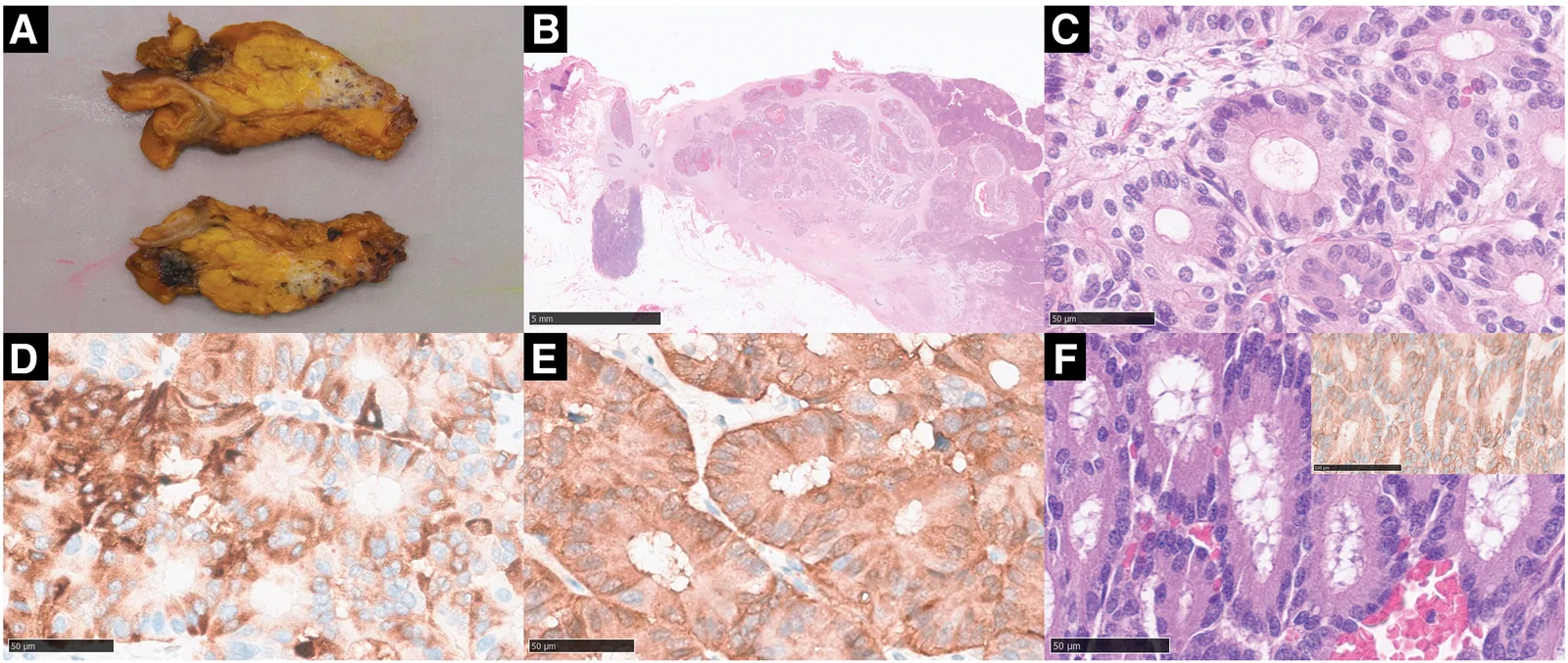
Resected pancreatic specimen. The tumor in the uncinate process of the pancreas was 25 × 16 mm in size, and the cut surface was grayish white (**A**). A low-power view showing pushing borders in sclerotic stroma (**B**). A high-power view showing tumor cells forming acinar structures with basally oriented nuclei (**C**). Immunohistochemically tumor cells are positive for synaptophysin (not shown), chromogranin A (**D**), and PP (**E**). The tumor in the hepatectomy specimen was initially diagnosed as iCCA but proved to be a PanNET with an acinar pattern (**F**), positive for PP (inset). iCCA, intrahepatic cholangiocarcinoma; PanNET, pancreatic neuroendocrine tumor; PP, pancreatic polypeptide

After 2 cycles of NAC-GS, CECT showed no remarkable change in tumor size, and pancreatoduodenectomy was performed. On POD 11, emergency arterial embolization was performed because of bleeding from the gastroduodenal artery caused by a postoperative pancreatic fistula (International Study Group on Pancreatic Fistula Grade C). No other complications were observed, and the patient was discharged on POD 45. The surgically resected specimen showed a 25 × 16-mm tumor with a grayish-white cut surface (**Fig. 4A**). Histological evaluation revealed an infiltrative pure acinar architecture (**Fig. 4B** and **4C**). Despite its acinar structure, immunohistochemical examination revealed positivity for synaptophysin, chromogranin A, CK18, SSTR2, and PP, was only scarcely positive for the exocrine markers CA19-9 and CEA, and was negative for CK7, CK20, and acinar cell markers such as trypsin and BCL10 (**Fig. 4D** and **4E**). Mitosis was 4/2 mm^2^ and the Ki-67 labeling index was 6%; therefore, the patient was diagnosed with a G2 PanNET according to the 2022 World Health Organization Classification.^7)^ The previously resected liver tissue from the referring hospital and the EUS-TA specimen were re-evaluated. The tumor cells in the liver, showing pure acinar structure, were also positive for neuroendocrine markers and PP (**Fig. 4F**). Therefore, the diagnosis was revised to hepatic metastases of the PanNET. Follow-up was performed every 3 months, and no evidence of pancreatic recurrence was observed for more than 18 months after surgery. The hepatic lesions were initially not resected because no progression was observed; however, elective surgical resection was scheduled because of favorable recovery.

## DISCUSSION

Radiological and pathological findings are essential for the diagnosis of PanNET. PanNETs usually show characteristic imaging and neuroendocrine histological features that allow differentiation from other pancreatic tumors. However, unusual findings can occur and may challenge the preoperative diagnosis, potentially leading to inappropriate treatment selection.

EUS-TA is a cornerstone of the preoperative diagnosis of pancreatic tumors. PanNETs usually exhibit solid and/or trabecular growth patterns.^8)^ Glandular patterns occasionally coexist with trabecular patterns derived from cell cords; however, pure glandular patterns are extremely rare. Notably, in a previously reported series of 185 PanNET cases, no case exhibited a pure acinar pattern, as observed in the present case.^9)^ Immunohistochemical analysis of PanNETs demonstrates positivity for neuroendocrine markers, including synaptophysin and chromogranin A.^10)^ As summarized in **Table 1**,^11–14)^ EUS-TA demonstrates high sensitivity, specificity, and accuracy for pancreatic tumors, including PanNETs. Nevertheless, diagnostic pitfalls may arise when tumors exhibit unusual morphological features that are not readily recognized as neuroendocrine. In the present case, EUS-FNA demonstrated a glandular and acinar pattern, which is uncommon for PanNETs, leading to misinterpretation as adenocarcinoma. The acinar architecture also raises the differential diagnosis of acinar cell carcinoma and mixed neuroendocrine–non-NENs. In the present case, these possibilities were not initially suspected because of the limited biopsy material and the clinical context. However, the resection specimen demonstrated diffuse expression of neuroendocrine markers, without evidence of acinar cell or exocrine differentiation, consistent with the diagnosis of PanNET. A retrospective review of the initial EUS-FNA specimen provided insight into the diagnostic challenges of this case. Although the EUS-FNA specimen provided sufficient material for histological examination and ancillary immunohistochemical studies, the fragmented nature of the biopsy restricted the assessment of the overall cytologic and architectural uniformity of the lesion. Consequently, neuroendocrine differentiation was not strongly suspected during the initial evaluation. Additional immunohistochemical studies performed at that time showed negativity for S100P and CRP and positivity for CD56. In retrospect, CD56 expression may have represented an early clue to neuroendocrine differentiation. However, this finding was not considered sufficient to prompt neuroendocrine marker studies because the morphology was interpreted within the framework of pancreaticobiliary adenocarcinoma, and the patient’s previous diagnosis of iCCA further reinforced that interpretation. Thus, the unusual histology, the limitations inherent to EUS-FNA sampling, and anchoring bias related to the previous diagnosis of iCCA collectively contributed to the failure to include neuroendocrine immunostaining in the initial workup. Taken together, careful clinicopathological correlation is essential, and repeated tissue acquisition or additional immunohistochemical evaluation to assess alternative diagnoses should be considered when biopsy findings are discordant with clinical or radiologic features.

**Table 1:** Diagnostic performance of EUS-TA

Parameter	All pancreatic tumors			PanNET
Source	Yang et al., 2016^11)^	Banafea et al., 2016^12)^	Crinò et al., 2021^14)^	Ito et al., 2017^13)^
Sensitivity	84%	90.8%	96.0%–97.3%	82.6%–100%
Specificity	98%	96.5%	100%	NR
Positive predictive value	NR	NR	100%	NR
Negative predictive value	NR	NR	46.2%–60.0%	NR
Accuracy	NR	91.0%	96.4%–97.4%	83.3%–93%

EUS-TA, endoscopic US-guided tissue acquisition; NR, not reported; PanNET, pancreatic neuroendocrine tumor

Radiological findings were also unusual and suggestive of PDAC. CT is a useful imaging modality for identifying PanNETs, with reported sensitivity and specificity of 67% and 83%, respectively.^15)^ PanNETs are typically well circumscribed and hypervascular on CECT and are often negative on ^18^F-FDG PET-CT.^16,17)^ However, ^18^F-FDG PET-CT is optional for higher-grade NENs, such as G3 NETs and NECs, because of higher glucose metabolism and lower somatostatin receptor expression.^10,18)^ Furthermore, some PanNETs may exhibit radiological findings similar to those of PDAC; they are often poorly circumscribed and hypovascular on CECT and positive on ^18^F-FDG PET-CT.^16,19)^ In this case, PDAC was suspected based on a poorly circumscribed hypovascular mass on CECT with FDG uptake on ^18^F-FDG PET-CT; however, G2 and G3 PanNETs should also have been considered in the differential diagnosis. The treatment strategy for resectable PDAC (R-PDAC) remains a subject of international debate. While NAC, such as the GS regimen, is currently recommended for R-PDAC in Japan,^5)^ there is no global consensus on the superiority of neoadjuvant therapy over upfront surgery for R-PDAC.^20)^ In cases where the preoperative diagnosis of adenocarcinoma is not entirely definitive, as in the present case, upfront surgery might be a valid consideration to avoid unnecessary toxicity and potential treatment delays caused by neoadjuvant therapy for a non-adenocarcinoma lesion.

From a retrospective viewpoint, a more cautious preoperative diagnostic assessment may have reduced the risk of diagnostic discrepancy. First, the unusually long survival after hepatectomy for metastatic iCCA should have prompted reconsideration of the pathological diagnosis and early re-evaluation with neuroendocrine immunohistochemical staining. Resectable iCCA accounts for only 20%–30% of all cases,^21)^ and even after curative surgical resection, the median and 5-year OS are approximately 28 months and 30%, respectively.^22)^ Furthermore, the presence of multiple hepatic tumors is associated with worse survival in iCCA,^23)^ with the median and 5-year OS reported to be <2 years and 17%–28%, respectively (**Table 2**).^24–26)^ Given this generally poor prognosis, survival for >3 years after surgical resection appears to be unusual. Second, when radiological findings are suggestive of PDAC but not entirely typical, high-grade PanNET should be considered in the differential diagnosis because treatment strategies differ substantially. If PanNET had been suspected preoperatively, additional functional imaging, including ^68^Ga/^64^Cu-DOTA-SSA PET-CT, might also have been considered to improve diagnostic accuracy, refine staging, and help evaluate eligibility for PRRT and SSA therapy. Third, atypical EUS findings might have provided an opportunity to reconsider the preoperative diagnosis. In the present case, EUS revealed a cystic lesion and no enhancement of the solid component, which was atypical for PDAC and raised the possibility of a cystic pancreatic neoplasm. However, at the multidisciplinary conference, the EUS-TA and CT/PET-CT findings were prioritized and considered more consistent with PDAC. Because EUS findings can provide important diagnostic information, discordance between EUS imaging and EUS-TA pathology should prompt careful reassessment of the biopsy interpretation, including pathological re-evaluation and additional immunohistochemical studies.

**Table 2:** Survival outcomes after resection of iCCA with multiple tumors

Source	Study type	Data source	Study period	Sample size, N	Median OS, months	5-year OS, %
Ribero et al., 2012^24)^	Prospective study	16 centers in Italy	1990–2008	140	NR	20.6
Spolverato et al., 2015^25)^	Retrospective study	11 international institutions	1990–2013	169	NR	17
Buettner et al., 2019^26)^	Retrospective study	12 international HPB institutions	1990–2017	185	21.2 (2 tumors) 15.3 (≥3 tumors)	28.0 (2 tumors) 8.6 (≥3 tumors)

HPB, hepatopancreatobiliary; iCCA, intrahepatic cholangiocarcinoma; NR, not reported; OS, overall survival

## CONCLUSIONS

We encountered a case of an unusual PanNET. The radiological findings were similar to those of PDAC, and the pathological findings included glandular and acinar histology. Multidisciplinary evaluation integrating the clinical course, imaging findings, and pathological assessment is essential to avoid unnecessary or suboptimal treatment.

## DECLARATIONS

### Availability of data and materials

The dataset supporting the conclusions of this study is included in the article.
